# Update and reuse: Structure-guided nanobody evolution against SARS-CoV-2 escape

**DOI:** 10.1371/journal.ppat.1014223

**Published:** 2026-05-18

**Authors:** Fan Bu, Divyasha Saxena, Hailey Turner-Hubbard, Amy Delaney, Lalit Batra, Charlie Fricke, Skyler Moye, Abhishek Verma, Stanley Perlman, Janarjan Bhandari, Bin Liu, Gang Ye, Jian Zheng, Fang Li

**Affiliations:** 1 Department of Pharmacology, University of Minnesota Medical School, Minneapolis, Minnesota, United States of America; 2 Center for Emerging Viruses, University of Minnesota, Minneapolis, Minnesota, United States of America; 3 Center for Predictive Medicine, University of Louisville, Kentucky, United States of America; 4 Department of Microbiology and Immunology, University of Iowa, Iowa City, Iowa, United States of America; 5 Hormel Institute, University of Minnesota, Austin, Minnesota, United States of America; 6 Department of Microbiology and Immunology, University of Louisville, Kentucky, United States of America; The Scripps Research Institute, UNITED STATES OF AMERICA

## Abstract

SARS-CoV-2 continues to accumulate spike mutations that erode the efficacy of antibody therapeutics. The Q493E mutation in the spike RBD, present in recent Omicron subvariants, enables escape from many antibodies and nanobodies, including our Nanosota-9A nanobody, which neutralizes Omicron JN.1 (Q493) but not KP.3 (E493). To address this, we applied a structure-guided in vitro evolution strategy to engineer Nanosota-9A, generating Nanosota-9B, which binds the KP.3 RBD with high affinity but shows reduced binding to JN.1 RBD. To regain breadth, we engineered a bispecific nanobody combining Nanosota-9A and -9B, which effectively neutralizes both JN.1 and KP.3 in infection assays. Our results provide proof of concept for an “update and reuse” strategy: applying structure-guided engineering to update and reuse validated nanobodies to overcome variant escape. This strategy offers a practical path to maintain therapeutic coverage as the virus evolves, supporting more efficient use of research resources and faster responses to emerging variants.

## Author summary

SARS-CoV-2 continues to acquire mutations that erode the effectiveness of existing antibody therapeutics, leading to repeated and resource-intensive discovery cycles. In this study, we developed a structure-guided in vitro evolution strategy to rapidly update a validated nanobody against an escape mutation in the spike receptor-binding domain. Instead of restarting discovery for each new variant, we reused an existing nanobody scaffold together with prior structural insights to generate an updated inhibitor through a streamlined engineering workflow. By introducing targeted changes, we restored binding to the escape variant and then combined the original and updated nanobodies into a bispecific molecule that neutralized multiple variants. This approach provides a practical way to maintain therapeutic coverage as the virus evolves, while conserving research resources and shortening response times. More broadly, these findings provide proof of concept for an updating strategy that may be useful for other rapidly mutating viruses in which escape mutations compromise antibody therapies, providing a template for durable, updatable antiviral biologics.

## Introduction

The COVID-19 pandemic highlighted a core vulnerability of antibody-based antivirals: once escape variants emerge, therapies can rapidly lose efficacy and be withdrawn, wasting prior investment and delaying care while new leads are rediscovered and redeveloped [1–3]. This outcome is predictable: during widespread transmission, mutations accumulate in the viral surface glycoproteins that mediate host-cell entry, undermining the practical utility of antibody drugs [4–6]. Yet antibodies retain key advantages over small molecules: they bind viral glycoproteins with high specificity, have favorable safety profiles, and possess long in vivo half-lives that often enable single-dose regimens [7,8]. Preserving these strengths while preventing viral escape will be essential in future pandemics. Here, we pursue that goal through structure-guided evolution of nanobodies.

Nanobodies are single-domain antibodies derived from camelid heavy-chain–only antibodies [9–11]. Their compact, modular architecture confers several antiviral advantages over conventional antibodies: access to cryptic epitopes and favorable tissue penetration; high in vitro stability that reduces cold-chain dependence; compatibility with intranasal or pulmonary delivery; and a straightforward phage-display workflow [12–18]. We recently developed a structure-guided strategy to rapidly adapt nanobodies to viral escape variants [19]. This approach maps escape mutations at the antigen-nanobody interface, builds focused libraries by randomizing nanobody residues proximal to those changes, and selects binders to the mutant antigen via phage display. The resulting targeted evolution proceeds far faster, while achieving comparable effectiveness to natural immune adaptation. It exploits the engineering simplicity of nanobodies’ single-domain scaffolds, an advantage not matched by the two-chain architecture of conventional antibodies. As proof of concept, we restored binding and neutralization against two prior mutations in the SARS-CoV-2 spike glycoprotein [19], which mediates viral entry [20,21]. In the present study, we apply this strategy to counter a newly emerged, clinically relevant spike escape mutation.

**Fig 1:**
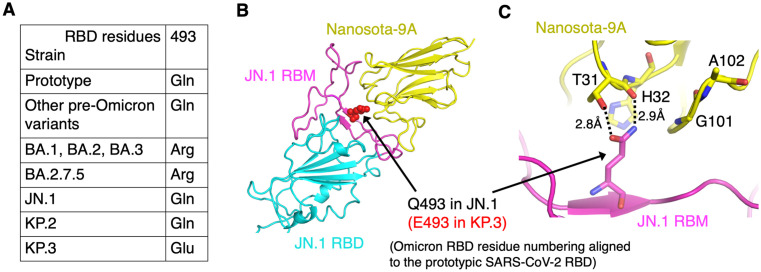
A single Q493E mutation in the Omicron JN.1 RBD abolishes Nanosota-9 binding. **(A)** Evolution at RBD residue 493 across SARS-CoV-2 variants and Omicron subvariants. **(B)** Structure of the JN.1 RBD bound to Nanosota-9A (PDB 9CO8). The JN.1 RBD core is shown in cyan, the receptor-binding motif (RBM) in magenta, and Nanosota-9A in yellow. Omicron RBD numbering is aligned to the prototypic SARS-CoV-2 RBD, and this numbering is used throughout this study. Residue 493 is shown as red sticks. **(C)** Detail of the JN.1 RBD-Nanosota-9A interface. Gln493 in the JN.1 RBD forms two hydrogen bonds: one with the Thr31 side chain and one with the main-chain carbonyl oxygen of His32 in Nanosota-9A. The Q493E mutation introduces a clash with the His32 main-chain carbonyl, abolishing Nanosota-9A binding to the RBD. Four nanobody residues near residue 493 (Thr31, His32, Gly101, and Ala102) were selected for randomization in the structure-guided in vitro evolution of Nanosota-9A.

The Omicron variant of SARS-CoV-2 emerged in late 2021 and rapidly displaced previous variants worldwide due to increased transmissibility (S1 Fig.) [22,23]. The earliest Omicron subvariants, BA.1, BA.2, and BA.3, were first detected in quick succession; BA.2 became dominant as BA.1 and BA.3 waned. BA.2 has continued to diversify, and recent subvariants (e.g., KP.2, KP.3, XFG) descend from JN.1, a BA.2 derivative [24,25]. During this evolution, the spike accumulated numerous mutations, many of which conferred antibody escape [26,27]. A recent mutation, Q493E in the receptor-binding domain (RBD) of KP.3 and XFG spikes, is particularly concerning (Fig 1A). In prototypic SARS-CoV-2, Gln493 forms two strong hydrogen bonds with Lys31 and Glu35 of the human ACE2 receptor [28,29]. Early Omicron subvariants replaced this residue with Arg493, a change incompatible with human Lys31 but compatible with Asn31 in mouse ACE2, consistent with the rodent-origin hypothesis for Omicron that we proposed [30]. Later subvariants, including JN.1, reverted to Gln493, restoring affinity for human ACE2 [31]. By contrast, Glu493 in KP.3 and XFG introduces charge repulsion with human Glu35, lowering the affinity of the interaction between the RBD and ACE2 and aligning with immune escape [27]. Because many RBD-directed antibodies target this region, the Q493E mutation is clinically significant [26,27].

To date, we have developed nine nanobodies - the Nanosota series - targeting the SARS-CoV-2 spike [15,32–34]. Nanosota-9 binds an RBD epitope that includes residue 493. It potently neutralizes JN.1 by directly blocking RBD-ACE2 binding but loses activity against KP.3 because of the Q493E mutation (Fig 1B, 1C) [33]. Here, we used our structure-guided in vitro evolution platform to update Nanosota-9 against this escape mutation. More broadly, this work provides proof of concept for an “update and reuse” strategy: applying structure-guided engineering to update and reuse validated nanobodies to counter newly emerged viral variants.

## Results

### Design and application of a structure-guided in vitro evolution strategy for Nanosota-9

We previously determined the cryo-EM structure of the JN.1 spike ectodomain complexed with Nanosota-9 [33]. The structure shows that two Nanosota-9 molecules cross-interact with two RBDs within the trimeric spike - one RBD in the “standing-up” (ACE2-accessible) state and the other RBD in the “lying-down” (immune-evasive) state (S2A Fig., S2B Fig.). The RBD contains two subdomains: a core and the receptor-binding motif (RBM), which mediates ACE2 engagement [20,28,35]. One Nanosota-9 binds the RBM of the standing-up RBD and directly blocks ACE2 binding. The other binds the core of the lying-down RBD, stabilizes it in the lying-down conformation, and indirectly blocks ACE2 binding. This cross-interacting mechanism underlies the high neutralization potency of Nanosota-9 against Omicron. Notably, the RBM epitope targeted by Nanosota-9 is relatively conserved compared with other RBD epitopes because of its essential role in ACE2 binding, which helps explain Nanosota-9’s broad anti-Omicron activity: it effectively neutralizes most Omicron subvariants, including JN.1 and KP.2 (S2C Fig.). However, this activity is disrupted by the Q493E mutation, and Nanosota-9 fails to neutralize KP.3 despite retaining activity against most other Omicron subvariants (Q489 in JN.1; E485 in KP.3) [33]. For consistency, we refer to this site as residue 493 (as in prototypic SARS-CoV-2) throughout, despite numbering differences among Omicron subvariants arising from spike insertions and deletions. This convention (i.e., using residue numbering from the prototypic SARS-CoV-2 spike as the reference) also applies to other RBD residues discussed below. Accordingly, our goal was to engineer Nanosota-9 to overcome the Q493E mutation in the KP.3 RBD.

**Fig 2:**
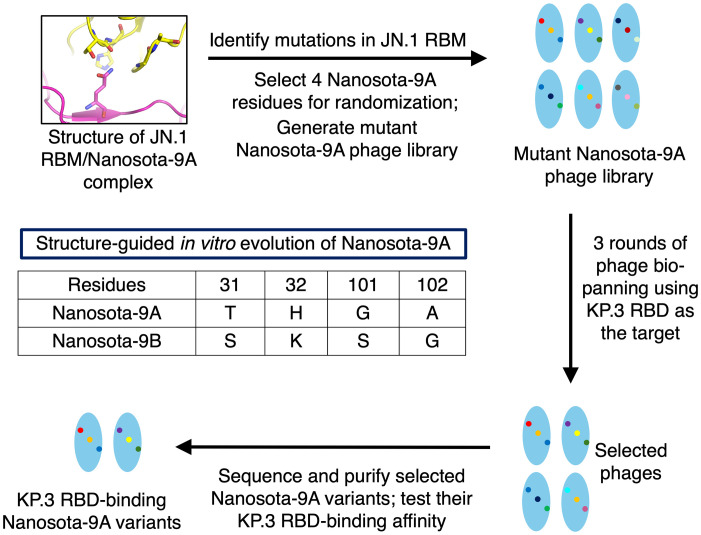
Flowchart of structure-guided in vitro evolution of Nanosota-9A to overcome the Q493E escape mutation in the KP.3 RBD. The workflow is shown along with the four nanobody residues selected for randomization, before (Nanosota-9A) and after the procedure (Nanosota-9B).

Our strategy was to apply our structure-based in vitro evolution approach to Nanosota-9 (Fig 2). At the JN.1 RBD-Nanosota-9 interface, Gln493 of the JN.1 RBD forms two hydrogen bonds with Nanosota-9: one with the Thr31 side chain and the other with the main-chain carbonyl oxygen of His32 (Fig 1B, 1C). In the KP.3 RBD, residue 493 is a Glu, which would be incompatible with the main-chain carbonyl oxygen of His32 in Nanosota-9. This incompatibility suggested that local remodeling of the nanobody interface would be required to accommodate Glu493. Therefore, to overcome this mutation, we targeted four nearby nanobody residues - Thr31, His32, Gly101, and Ala102 - for simultaneous randomization. These randomized changes were introduced into the gene encoding Nanosota-9, now termed Nanosota-9A, in the phage vector using PCR primers. The mutant Nanosota-9A phage display library was then constructed and screened with the KP.3 RBD as bait. Phages that bound to the KP.3 RBD were sequenced to identify the mutant nanobody genes they carried. Screening identified two mutant nanobodies with enhanced affinity for the KP.3 RBD: one containing Ser31/Lys32/Ser101/Gly102, and the other containing His31/Leu32/deletion101/deletion102 (S3A Fig.). Because the former showed higher affinity for the KP.3 RBD based on ELISA (S3B Fig.), it was selected for further characterization and designated Nanosota-9B.

### Functional characterization of Nanosota-9B

**Fig 3:**
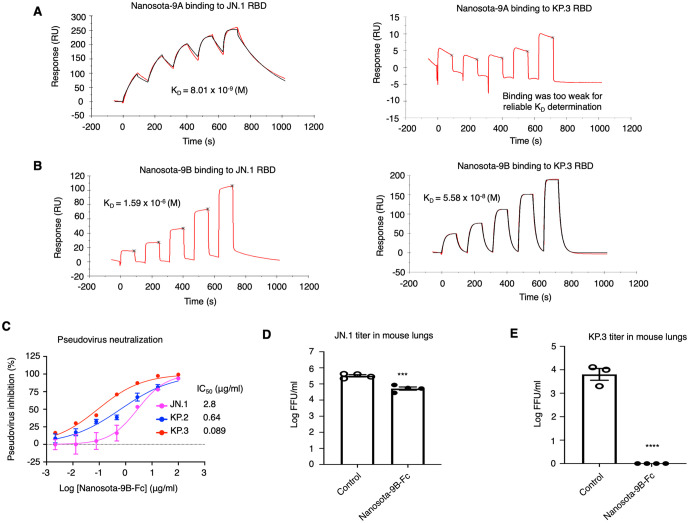
Functional characterization of Nanosota-9B. **(A)** Surface plasmon resonance (SPR) analysis of binding between Nanosota-9A and Omicron RBDs. For each measurement, a His-tagged RBD (JN.1 or KP.3) was immobilized on a CM5 sensor chip, and His-tagged Nanosota-9A was used as the analyte. Nanosota-9A bound the JN.1 RBD with high affinity but bound the KP.3 RBD too weakly to allow reliable determination of K_D. **(B)** SPR analysis of binding between Nanosota-9B and Omicron RBDs. For each measurement, a His-tagged RBD (JN.1 or KP.3) was immobilized on a CM5 sensor chip, and His-tagged Nanosota-9B was used as the analyte. Nanosota-9B bound the KP.3 RBD with high affinity but bound the JN.1 RBD with lower affinity. **(C)** Efficacy of Nanosota-9B-Fc in neutralizing Omicron pseudoviruses. Retroviruses pseudotyped with Omicron spike (from JN.1, KP.2, or KP.3) were used to infect human ACE2-expressing cells in the presence of Nanosota-9B-Fc at various concentrations. Entry efficiency was measured by luciferase signal. The efficacy of Nanosota-9B-Fc against each pseudovirus type was expressed as the concentration capable of neutralizing pseudovirus entry by 50% (IC_50_). Error bars represent SEM (n = 3). Each experiment was repeated at least three times with similar results. **(D)** and **(E)** Efficacy of Nanosota-9B-Fc in neutralizing authentic JN.1 **(D)** and KP.3 **(E)** in mice. C57BL/6 mice were challenged by intranasal inoculation with Omicron subvariants. Nanosota-9B-Fc was administered at 10 mg/kg body weight 4 hours post-infection. In the treatment group (n = 4 for each virus), mice received Nanosota-9B-Fc intraperitoneally. In the control group (n = 4 for JN.1 and n = 3 for KP.3), mice received PBS. Lung virus titers on day 2 post-infection were measured by focus-forming assay (FFA). Comparisons of lung virus titers between control and treatment groups were performed using an unpaired two-tailed Student’s t-test. *** p < 0.001; **** p < 0.0001.

We evaluated the anti-Omicron activities of Nanosota-9B, focusing on JN.1 and KP.3. First, we measured the binding of Nanosota-9A and Nanosota-9B (both as His-tagged monomers) to the JN.1 and KP.3 RBDs (also as His-tagged monomers) using surface plasmon resonance (SPR). Nanosota-9A bound the JN.1 RBD with high affinity (K_D_ = 8.01 nM) but its binding to the KP.3 RBD was too weak for reliable K_D_ determination (Fig 3A), consistent with our earlier report [33]. In contrast, Nanosota-9B bound the KP.3 RBD with high affinity (K_D_ = 55.8 nM) but bound the JN.1 RBD with lower affinity (K_D_ = 1.59 μM) (Fig 3B). Thus, engineering Nanosota-9A to Nanosota-9B restored binding to the KP.3 RBD at the cost of reduced binding to the JN.1 RBD. Second, we assessed the potency of Nanosota-9B in neutralizing Omicron spike-mediated entry. We performed an Omicron pseudovirus entry assay in which HIV pseudotyped with an Omicron spike infected ACE2-expressing human cells in the presence of human Fc-tagged Nanosota-9B (Nanosota-9B-Fc). Nanosota-9B-Fc neutralized KP.3 pseudoviruses more efficiently than JN.1 pseudoviruses, with IC_50_ values of 0.089 μg/mL and 2.8 μg/mL, respectively (Fig 3C). It also neutralized KP.2 pseudoviruses with an IC_50_ of 0.64 μg/mL. This also contrasts with Nanosota-9A, which potently neutralizes JN.1 pseudoviruses but does not neutralize KP.3 pseudoviruses [33]. Together, these data demonstrate that Nanosota-9B targets KP.3 significantly more efficiently than JN.1, while retaining meaningful activity against JN.1. In contrast, Nanosota-9A potently targets JN.1 but not KP.3.

We further evaluated the anti-Omicron potency of Nanosota-9B in mice. We used the Fc-tagged form (Nanosota-9B-Fc) for several reasons. Fc tagging typically enhances antiviral potency by increasing valency and potentially inducing antibody-dependent cellular cytotoxicity (ADCC). In addition, with a molecular weight of ~75 kDa, Fc-tagged nanobodies have prolonged in vivo half-life (typically 5–10 days) due to both reduced renal clearance associated with increased molecular size and neonatal Fc receptor (FcRn)–mediated recycling [36]. Despite the added mass, Fc-tagged nanobodies are still about half the size of conventional antibodies, remain compatible with intranasal delivery, and retain high in vitro stability. Importantly, they preserve the single-domain antigen-binding architecture. For in vivo efficacy testing, C57BL/6 mice were challenged with either JN.1 or KP.3. Four hours after infection, Nanosota-9B-Fc was administered by intraperitoneal injection. Lung tissues were collected 2 days post-infection for viral titer measurement, which is a commonly used timepoint for assessing antiviral efficacy in Omicron mouse models, where infection is typically mild and lung viral titers provide the most reliable readout [33,34]. Nanosota-9B-Fc reduced JN.1 titers in the lungs by six-fold, and reduced KP.3 titers to undetectable levels (Fig 3D, 3E). Consistent with the in vitro data, these in vivo results show that Nanosota-9B-Fc provides complete protection against KP.3 infection and only modest protection against JN.1.

### Structural characterization of Nanosota-9B

**Fig 4:**
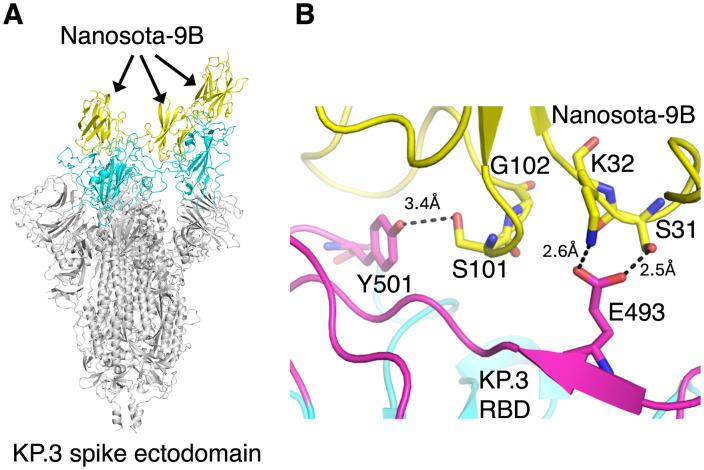
Structure of the KP.3 spike ectodomain complexed with Nanosota-9B. **(A)** Overall cryo-EM structure of the KP.3 spike ectodomain complexed with Nanosota-9B. The KP.3 spike ectodomain is shown in gray, with the three RBDs in cyan. One RBD is in the standing-up conformation and two are in the lying-down conformation. Nanosota-9B (yellow) binds all three RBDs. **(B)** Detailed view of the KP.3 RBD-Nanosota-9B interface. The newly evolved nanobody residues Ser31 and Lys32 form a hydrogen bond and a salt bridge, respectively, with Glu493 of the KP.3 RBD, overcoming the Q493E mutation. Another newly evolved nanobody residue, Ser101, forms a hydrogen bond with Tyr501 of the KP.3 RBD, further stabilizing the KP.3 RBD-Nanosota-9B interface.

To elucidate the structural basis for Nanosota-9B’s high-affinity binding to the KP.3 spike, we determined the cryo-EM structure of the KP.3 spike ectodomain complexed with Nanosota-9B (S4 Fig.; S1 Table). Nanosota-9B engages the trimeric KP.3 spike in the same manner as Nanosota-9A engages the trimeric JN.1 spike: Nanosota-9B binds all three RBDs, with one RBD in the standing-up state and two in the lying-down state; among these, two Nanosota-9B molecules cross-interact with two RBDs, with one RBD in the standing-up state and the other in the lying-down state (Fig 4A). The structure reveals a modified interaction network at the KP.3 RBD-Nanosota-9B interface, arising from substitutions in both the RBD and the nanobody (Fig 4B). Specifically, Glu493 in the KP.3 RBD forms a hydrogen bond with Ser31 and a salt bridge with Lys32 of Nanosota-9B. The main-chain carbonyl of Thr31 in Nanosota-9A, which previously conflicted with Glu493, is now reoriented and no longer clashes with this residue. Although the additional substitutions in Nanosota-9B at residues 101 and 102 do not directly accommodate Glu493, Ser101 forms a hydrogen bond with Tyr501 in the KP.3 RBD, further strengthening the interface. These structural analyses define how Nanosota-9B accommodates Glu493 and achieves high-affinity binding to the KP.3 RBD.

Superposition of Nanosota-9A and Nanosota-9B shows that their overall structures are highly similar, with differences confined to the engineered residues. In particular, substitutions at positions 101 and 102, which include changes from or to glycine, induce small local conformational changes in the corresponding loop (S5 Fig.). By contrast, substitutions at positions 31 and 32 do not cause significant local conformational changes in the corresponding loop. Overall, the structural changes resulting from engineering are minimal, in contrast to our previous engineering of Nanosota-3A, which involved more significant structural changes [19].

### Construction and characterization of bispecific nanobody Nanosota-9A/9B-Fc

**Fig 5:**
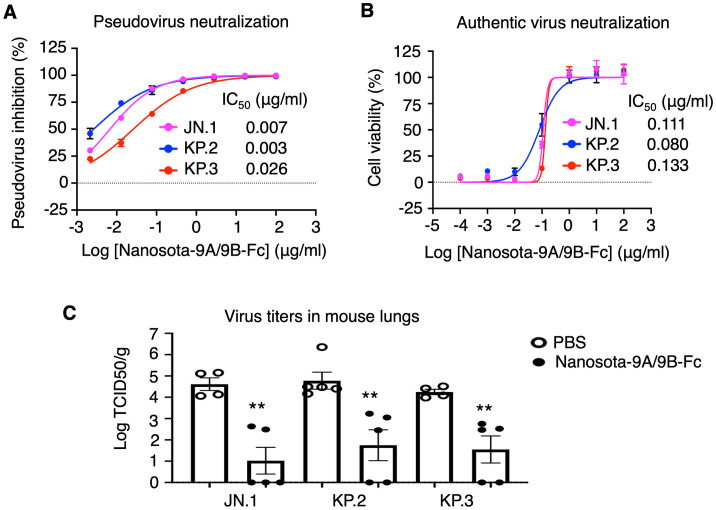
Construction and functional characterization of the bispecific nanobody Nanosota-9A/9B-Fc. A bispecific nanobody, Nanosota-9A/9B-Fc, was generated by fusing Nanosota-9A and Nanosota-9B to a human Fc domain. **(A)** Efficacy of Nanosota-9A/9B-Fc in neutralizing Omicron pseudoviruses. The assay was performed as in Fig 3C. **(B)** Efficacy of Nanosota-9A/9B-Fc in neutralizing authentic Omicron in vitro. Each Omicron subvariant infected Vero E6-ACE2-TMPRSS2 cells in the presence of Nanosota-9A/9B-Fc at different concentrations. Infection efficiency was determined by the remaining cell viability after 96 hours (lower viability indicates higher infection). Efficacy for each subvariant is expressed as the IC₅₀, the concentration that reduces virus-induced cytopathic effect by 50% relative to serum-exposed virus controls. Data are mean ± SEM (n = 4). **(C)** Efficacy of Nanosota-9A/9B-Fc in neutralizing authentic Omicron (JN.1, KP.2, and KP.3) in mice. The experiment followed Fig 3C and Fig 3D, except lung virus titers on day 2 post-infection were measured by TCID₅₀ assay. Treatment groups, n = 5; control groups, n = 4. Comparisons of lung virus titers between the control and treatment groups were performed using an unpaired two-tailed Student’s t-test. **p < 0.01.

In a previous study, we developed Nanosota-9A, which potently inhibits JN.1 but not KP.3, and in the current study we developed Nanosota-9B, which potently inhibits KP.3 but shows only moderate activity against JN.1. To create a single inhibitor effective against both Omicron JN.1 and KP.3 subvariants, we constructed the bispecific nanobody Nanosota-9A/9B-Fc by fusing Nanosota-9A and Nanosota-9B to a human Fc domain. In vitro, Nanosota-9A/9B-Fc potently neutralized JN.1, KP.2, and KP.3 pseudoviruses with IC_50_ values of 0.007, 0.003, and 0.026 μg/mL, respectively (Fig 5A). It also neutralized the corresponding authentic viruses with IC_50_ values of 0.111, 0.080, and 0.133 μg/mL, respectively (Fig 5B). For in vivo testing, C57BL/6 mice were challenged with JN.1, KP.2, or KP.3, treated intraperitoneally with Nanosota-9A/9B-Fc 4 hours after infection, and assessed for lung viral titers 2 days later; the bispecific nanobody significantly reduced titers for all three subvariants by approximately 10^3^ to 10^5^-fold (Fig 5C). These results show that Nanosota-9A/9B-Fc is a potent inhibitor of JN.1, KP.2, and KP.3 in vitro and in vivo.

We next tested the neutralizing potency of Nanosota-9A/9B-Fc against XFG, the dominant Omicron subvariant in the United States (85% as of September 2025) [37], and found that it did not neutralize XFG pseudoviruses (S6A Fig.). Sequence alignment of Omicron RBDs identified four substitutions between JN.1 and XFG in or near the Nanosota-9 epitope: R346T and H445R (proximal to the epitope) and F456L and Q493E (within the epitope) (S6B Fig., S6C Fig.). Because Nanosota-9B accommodates the Q493E mutation (present in KP.3) and Nanosota-9A accommodates the F456L mutation (present in KP.2) and the R346T mutation (present in XBB.1.5), the loss of Nanosota-9A/9B-Fc activity against XFG is most likely attributable to the H445R mutation, despite its location outside the epitope. Addressing this mutation will be the focus of future work, whereas the present study expands our Nanosota arsenal by overcoming the Q493E mutation and provides proof of concept for our proposed “update and reuse” strategy.

## Discussion

Rapid escape mutations in respiratory RNA viruses such as SARS-CoV-2 expose a core limitation of conventional antibody therapeutics: an antibody’s paratope (its antigen-binding site) can fail abruptly when a single escape mutation on the viral target abolishes binding, forcing slow, repeated, and resource-intensive discovery cycles. A durable antiviral platform must therefore either target conserved viral epitopes or enable swift, rational adaptation to viral-variant-specific mutations. In practice, the most potent SARS-CoV-2 antibodies target the spike RBD, whereas antibodies targeting non-RBD epitopes generally lack sufficient antiviral potency to advance to clinical use [38–41]. Therapies must therefore confront the evolutionarily dynamic RBD directly. We propose an “update and reuse” paradigm for nanobody therapeutics, using structure-guided in vitro nanobody evolution to counter key viral escape mutations and to continually expand antiviral arsenals. Nanobodies are well-suited to this approach because their single-domain architecture simplifies molecular cloning and bacterial expression in phage display workflows, whereas conventional two-chain antibodies complicate them. Nanobodies therefore offer a practical route to rapid, durable responses to current and future viral variants.

We apply this blueprint to a concrete Omicron RBD problem to demonstrate its practical relevance. Starting from a validated Nanosota-9A scaffold that is potent against JN.1 but impaired by the Q493E mutation in the KP.3 RBD, we mapped the mutation-induced clash at the RBD-nanobody interface and fully randomized four nanobody residues near the mutation site. Selection against the KP.3 RBD yielded Nanosota-9B, which overcomes the Q493E mutation by locally reorganizing around Glu493 and forming new stabilizing contacts with the KP.3 RBD. Functionally, Nanosota-9B binds and neutralizes KP.3 more effectively than JN.1 while retaining moderate JN.1 activity. A bispecific nanobody combining Nanosota-9A and Nanosota-9B potently neutralizes JN.1, KP.2, and KP.3 in vitro and in vivo. Because protein-protein interfaces are intrinsically plastic [42,43], multiple engineering solutions can defeat a given viral escape mutation, depending on which nearby nanobody residues are randomized. In prior work, for example, we re-engineered Nanosota-3A, active against BA.1 but not XBB.1.5, into Nanosota-3C, which targets both BA.1 and XBB.1.5 RBDs with high affinity, showing that adaptation to a new viral variant can preserve activity against the prior one [19]. Thus, for each recurrent viral escape mutation, multiple viral-variant-adapted nanobodies and the design rules behind them can be accumulated across epitopes on the SARS-CoV-2 spike, building coverage against a broad set of mutations. Starting from the basic case as shown in the current study, in which one nanobody addresses a single escape mutation within one epitope, this framework further generalizes in two ways: (i) adapting a single nanobody to multiple escape mutations within the same epitope and (ii) extending to panels of nanobodies that target distinct epitopes on the SARS-CoV-2 spike.

A central advantage of this proposed “update and reuse” strategy is cumulative reuse. Each successful adaptation contributes a reusable module: a viral-variant-adapted paratope, its structural rationale, and a validated set of residues for future randomization that can be redeployed or recombined as related mutations recur. This maximizes prior investment and speeds subsequent updates. By contrast, rediscovery cycles for conventional antibodies rarely carry antiviral efforts forward; newly selected antibodies replace rather than augment earlier antibodies and offer limited reusability when new viral variants emerge.

The membrane-fusion S2 subunit of SARS-CoV-2 spike is more conserved than its receptor-binding S1 subunit, including the RBD, and therefore represents an alternative antiviral target [20]. During entry, S2 transitions from the prefusion state to the postfusion state through an intermediate state [44]. Neutralizing antibodies and nanobodies targeting S2 have been reported [38–41]. However, despite its stronger conservation, S2 is generally a less favorable target for highly potent neutralization because S2 epitopes are less accessible in the prefusion spike and become exposed only in the intermediate state, which is transient and may occur in endosomes that antibodies do not easily access. Accordingly, S2-directed binders often illustrate a breadth-over-potency tradeoff: conservation of S2 can support broader reactivity, but typically with substantially lower neutralization potency than the strongest RBD-directed binders. Moreover, although structural studies of S2-directed binders have been reported, they generally rely on isolated small fragments corresponding to the intermediate-state S2 rather than full-length spike structures, making their mechanisms of neutralization less directly established. Therefore, our current strategy remains focused on the RBD, which is the principal target of highly potent neutralizing antibodies and nanobodies but is also vulnerable to escape mutations. In this context, the limited potency and less definitive mechanistic characterization of S2-targeting binders, together with the high potency but escape-prone nature of RBD-targeting binders, underscore the value of our strategy for pandemic preparedness.

Computational antibody redesign approaches have shown promise for restoring or broadening antiviral activity against SARS-CoV-2 escape variants [45,46]. These approaches allow large-scale in silico exploration of sequence space and rapid prioritization of candidate mutations. However, their performance depends on accurate structural modeling and predictive scoring, and can be substantially compromised when mutations induce structural rearrangements beyond local side-chain changes. In addition, their predictions still require experimental validation, which can slow the overall turnaround time. In contrast, our structure-guided in vitro evolution approach does not depend on accurate structural models or prediction of mutation-induced structural changes, because it experimentally surveys all possible combinations at selected nanobody positions near the escape site and directly selects functional binders. As a result, it provides a more direct and reliable route to identifying functional nanobody variants. A limitation of our approach is that it is currently best suited to nanobodies. Overall, computational redesign and structure-guided in vitro evolution represent complementary strategies for countering viral escape, with our approach offering clear advantages in directly capturing functional solutions without reliance on predictive modeling.

In sum, updating and reusing rather than rediscovering offers a practical way to keep pace in the arms race with viruses. It provides a rapid, repeatable, mechanistically grounded workflow; preserves and builds on prior work through modular arsenals of viral-variant-adapted nanobodies; and yields binders whose activity tracks newly dominant viral mutations. Although viral escape mutations are frequent, there are finite evolutionary pathways available to a virus. As surveillance highlights recurrent viral mutation hotspots, new designs of nanobodies can begin immediately using the accumulated knowledge base. While we demonstrate this updating workflow in two nanobody-variant contexts to date, broader generalization will benefit from additional applications across diverse epitopes and viral targets. This nanobody-centered, structure-guided approach provides proof of concept for a practical path to sustain antiviral coverage while avoiding the delays and wastefulness inherent to rediscovery.

## Methods

### Ethics statement

This study was performed in strict accordance with the recommendations in the *Guide for the Care and Use of Laboratory Animals* of the National Institutes of Health (NIH). All animals were handled according to approved institutional animal care and use committee (IACUC) protocols of the University of Louisville (protocol number: 22134) and the University of Iowa (protocol number: 9051795). All procedures involving SARS-CoV-2 were conducted in approved biosafety level 2+ or 3 facilities.

### Cell lines, plasmids and viruses

HEK293T and Vero E6-ACE2-TMPRSS2 cells (Vero E6 cells overexpressing human ACE2 and human TMPRSS2) (ATCC) were cultured in Dulbecco’s modified Eagle medium (DMEM) supplemented with 10% fetal bovine serum. TG1 and SS320 E. coli (Lucigen) were grown in 2YT medium. The prototypic SARS-CoV-2 spike gene (GenBank: QHD43416.1) was synthesized (GenScript) with the D614G substitution. The XFG spike gene (GISAID: EPI_ISL_20063407) was also synthesized (Twist Bioscience). Mutations were introduced into the prototypic spike gene to generate spike genes corresponding to the Omicron subvariants JN.1 (GISAID: EPI_ISL_17774216), KP.2 (GISAID: EPI_ISL_19214303), and KP.3 (GISAID: EPI_ISL_19214243). All spike genes were cloned into the pcDNA3.1(+) vector.

Genes encoding Omicron spike ectodomains (residues 1–1207 for JN.1 and 1–1203 for KP.3) were subcloned into the pCAGGS vector (Addgene) with a C-terminal His tag and foldon trimerization motif. In these constructs, the furin cleavage motif RRAR was replaced with AGAR, and six proline substitutions were introduced into the S2 subunit as described previously [47,48]. Genes encoding Omicron RBDs (residues 316–531 for JN.1 RBD and 316–531 for KP.3 RBD) were subcloned into the pCAGGS vector with a C-terminal His tag. Genes encoding Fc-tagged nanobodies were cloned into pCAGGS with an N-terminal tPA signal peptide and a C-terminal human IgG1 Fc (GenBank: AEV43323.1). To generate the bispecific nanobody Nanosota-9A/9B-Fc, a “knobs-into-holes” strategy was used, introducing T366Y and Y407T into the Fc regions of the pCAGGS-Nanosota-9A and pCAGGS-Nanosota-9B constructs, respectively [49].

Omicron isolates hCoV-19/USA/New York/PV96109/2023 (subvariant JN.1; BEI NR-59693), hCoV-19/USA/CA-GBW-GKISBBBB26982/2024 (subvariant KP.2; BEI NR-59890), and hCoV-19/USA/NJ-GBW-GKISBBBB88291/2024 (subvariant KP.3; BEI NR-59892) were obtained from BEI Resources, NIAID, NIH.

### Structure-guided in vitro evolution of Nanosota-9

To enhance binding to the KP.3 RBD, Nanosota-9A was subjected to structure-guided in vitro evolution as previously described [33]. Random mutations at Thr31, His32, Gly101, and Ala102 were introduced by PCR, generating a library of Nanosota-9A variants. The mutant nanobody genes were cloned into the pADL22c vector (Antibody Design Labs) and introduced into TG1 cells by electroporation to construct a mutagenic phage-display library. Phages with improved binding to the KP.3 spike ectodomain were then selected by bio-panning. After three rounds, the best-binding phages were identified, their nanobody genes sequenced, and the corresponding nanobodies tested for affinity for the KP.3 spike ectodomain by ELISA. The top binder, named Nanosota-9B, contains four mutations: T31S, H32K, G101S, and A102G.

### Protein expression and purification

Nanosota-9A and Nanosota-9B were expressed and purified as previously described [15]. Briefly, His- and HA-tagged nanobodies were purified from the periplasm of SS320 E. coli after induction with 1 mM IPTG. E. coli cells were harvested and resuspended in 15 mL TES buffer (0.2 M Tris, pH 8.0; 0.5 mM EDTA; 0.5 M sucrose). Proteins in the supernatant were purified sequentially using a Ni-NTA column and a Superdex 200 gel-filtration column (Cytiva).

The Omicron spike ectodomains (JN.1 and KP.3, each His-tagged), Omicron RBDs (JN.1 and KP.3, each His-tagged), and Fc-tagged nanobodies were prepared as previously described [33]. Plasmids were transiently transfected into Expi293F cells using polyethylenimine (PEI; Polysciences). Supernatants were harvested 3 days post-transfection. Proteins were purified using a Ni-NTA affinity column (Cytiva), followed by a Superose 6 gel-filtration column (Cytiva) for spike ectodomains or a Superdex 200 gel-filtration column (Cytiva) for Fc-tagged nanobodies.

### ELISA

ELISA was performed to measure the binding affinity between His-tagged Omicron spike ectodomains and HA-tagged nanobodies as previously described [15]. Briefly, ELISA plates were coated with a recombinant Omicron spike ectodomain and then sequentially incubated with nanobodies (either nanobodies from SS320 E. coli supernatant or recombinant nanobodies) and HRP-conjugated anti-HA antibody (1:2,000; Sigma). The ELISA substrate (Invitrogen) was added, the reaction was stopped with 1 N H₂SO₄, and absorbance at 450 nm was measured on a Synergy LX Multi-Mode Reader (BioTek).

### Surface plasmon resonance

Surface plasmon resonance (SPR) was performed to measure binding affinities between a nanobody (Nanosota-9A or Nanosota-9B, both His-tagged monomers) and an RBD (from JN.1 or KP.3, both His-tagged monomers) using a Biacore S200 system (Cytiva), as previously described with minor modifications [50,51]. Recombinant RBDs were immobilized on a CM5 sensor chip (Cytiva) by chemical cross-linking. Recombinant nanobodies were injected at various concentrations in running buffer containing 10 mM HEPES (pH 7.4), 150 mM NaCl, 3 mM EDTA, and 0.005% surfactant P20. Binding responses were recorded as response units (RU), and the data were analyzed using Biacore Evaluation Software (Cytiva).

### Pseudovirus entry assay

Pseudovirus entry assays were performed as previously described [33]. Pseudoviruses were generated by co-transfecting HEK293T cells with a pcDNA3.1(+) plasmid encoding a full-length spike, the helper plasmid psPAX2 (lentiviral backbone), and the luciferase reporter plasmid pLenti-CMV-luc. After 72 hours, pseudoviruses were collected, incubated with nanobodies at varying concentrations at 37 °C for 1 hour, and then used to infect HEK293T cells expressing human ACE2. After an additional 60 hours, cells were lysed. Aliquots of the lysates were transferred to new plates, luciferase substrate was added, and relative light units (RLUs) were measured on an EnSpire plate reader (PerkinElmer). Nanobody efficacy was reported as the IC₅₀, the concentration required to inhibit pseudovirus entry by 50%.

### Omicron microneutralization assay

The neutralizing potency of Nanosota-9A/9B-Fc against authentic Omicron infection was assessed by virus microneutralization as previously described [52]. Briefly, Nanosota-9A/9B-Fc was serially diluted 10-fold in DMEM starting at 100 µg/mL. Each dilution (in quadruplicate) was mixed with one Omicron subvariant (JN.1, KP.2, or KP.3) at an MOI of 0.01 and incubated at 37 °C for 45 minutes. Mixtures were added to Vero E6-ACE2-TMPRSS2 cells seeded the previous day in 96-well plates. After 1 hour, the inoculum was replaced with 1 × DMEM containing 5% FBS. Cell viability was measured after 96 hours using a Neutral Red assay (Sigma-Aldrich). Potency was reported as the IC₅₀ - the concentration required to reduce virus-induced cytopathic effect by 50% relative to virus-only controls.

### Evaluation of the anti-Omicron potency of Nanosota-9B-Fc in mice

C57BL/6 male mice (10 weeks old) were obtained from Charles River Laboratories. They were housed in groups of up to five, given free access to food and water, and kept on a 12-hour light/dark cycle. Mice were lightly anesthetized with ketamine/xylazine and intranasally challenged with 10^5^ PFU of the Omicron subvariant JN.1 or KP.3. Four hours after infection, mice received 10 mg/kg Nanosota-9B-Fc intraperitoneally. Clinical scores and body weight were monitored daily. All mice were euthanized on day 2 post-infection, and lungs were collected into DMEM and homogenized.

Virus titers were measured by focus-forming assay (FFA). Lung homogenate supernatants were diluted 10-fold in DMEM; 50 µL of each dilution was added to confluent Vero E6-ACE2-TMPRSS2 cells in flat-bottom 96-well plates and incubated for 45 minutes at 37 °C with 5% CO₂. The inoculum was replaced with 100 µL of overlay medium (10% FCS, penicillin/streptomycin, 1.2% carboxymethylcellulose). Plates were incubated for 20 hours at 37 °C, 5% CO₂. After fixation, cells were washed with 0.1% Tween-20/PBS, then permeabilized and blocked for 30 minutes at room temperature in 0.1% Triton X-100/1% BSA/PBS. Viral foci were stained with a mouse anti-SARS-CoV/SARS-CoV-2 nucleocapsid antibody (SinoBiological; 1:1,000 in PBS with 1% BSA), followed by an HRP-conjugated goat anti-mouse IgG (Invitrogen; 1:500 in PBS with 1% BSA) for 1 hour at 37 °C. Bound antibodies were detected with KPL TrueBlue Peroxidase Substrate (SeraCare) for 10 minutes at room temperature. Plates were rinsed with distilled water, air-dried, and imaged; foci were counted using a CTL ImmunoSpot Analyzer.

### Evaluation of the anti-Omicron potency of Nanosota-9A/9B-Fc in mice

The neutralizing potency of Nanosota-9A/9B-Fc against authentic Omicron in vivo was assessed in mice as previously described [33]. Briefly, female C57BL/6J mice (n = 5 per group) were challenged by intranasal inoculation with Omicron subvariants JN.1, KP.2, or KP.3 (10^4^ PFU per mouse) in 50 µL DMEM. Infected mice received either Nanosota-9A/9B-Fc (10 mg/kg body weight) or PBS by intraperitoneal injection 4 hours post-challenge. Mice were euthanized on day 2 post-infection; lungs were collected, homogenized, and stored at −80 °C until analysis.

Viral titers in lung tissue were determined by TCID₅₀ as previously described [52]. Vero E6-ACE2-TMPRSS2 cells were seeded in 96-well tissue-culture plates and incubated overnight at 37 °C. The next day, lung-homogenate supernatants were serially diluted 10-fold in growth medium (DMEM with 5% FBS) and added in quadruplicate to the plates. Cultures were incubated at 37 °C in 5% CO₂. At 4 days post-infection, cells were fixed in 10% neutral-buffered formalin and stained with 0.1% crystal violet to assess cytopathic effect. TCID₅₀ values were calculated using the Reed–Muench method.

### Cryo-EM grid preparation and data acquisition

KP.3 spike ectodomain was mixed with a 1.5-fold molar excess of Nanosota-9B for 1 h before grid preparation. Four microliters (0.8 mg/mL) of the mixture were applied to freshly glow-discharged Quantifoil R1.2/1.3, 300-mesh copper grids (Electron Microscopy Sciences), blotted for 4 s at 22 °C under 100% chamber humidity, and plunge-frozen in liquid ethane using a Vitrobot Mark IV (FEI). Cryo-EM data were collected in EPU (Thermo Fisher Scientific) with a K3 direct electron detector and a BioContinuum energy filter (Gatan) at a nominal magnification of 130,000× (0.664 Å/pixel) (S1 Table).

### Cryo-EM image processing

Cryo-EM data were processed in cryoSPARC v4.5.1 [53]. Dose-fractionated movies underwent Patch motion correction with MotionCor2 [54] and Patch CTF estimation with CTFFIND [55]. Particles were picked with the Blob Picker and Template Picker, followed by the Remove Duplicate Particles tool. Junk particles were discarded through three rounds of 2D classification. Particles from the good 2D classes were used for ab initio reconstruction of four maps, then subjected to 3D classification into six classes. The two best 3D classes were refined further with non-uniform and CTF refinements to generate the final maps. To improve density at the RBD-nanobody interface, local refinements focused on one lying-down RBD (the best-resolved of the three) and the Nanosota-9B region. Map resolutions were determined by gold-standard Fourier shell correlation (FSC) at 0.143 between the two half-maps. Local resolution was estimated from the half-maps in cryoSPARC v4.5.1 (S4 Fig.).

### Cryo-EM model building and refinement

Initial model building of the spike/nanobody complexes was performed in Coot v0.8.9 [56] using PDB 9CO8 as the starting model [33]. Models were refined through iterative cycles of Phenix v1.16 [57] and manual rebuilding in Coot until final, reliable models were obtained. Standing-up RBDs and their bound nanobodies are generally flexible; therefore, they were fitted into the density as rigid bodies. In the local map of the KP.3 spike/Nanosota-9B complex, an atomic model was built for the interface between the lying-down RBD and Nanosota-9B (S1 Table). Figs were generated in PyMOL (The PyMOL Molecular Graphics System, v3.0; Schrödinger, LLC).

## Supporting information

S1 Fig Schematic relationships among representative SARS-CoV-2 Omicron lineages [37]. This diagram is not intended as a phylogenetic tree. Shown are BA.1, BA.2, BA.3, BA.5/BQ.1, BA.2.7.5, XBB.1.5/EG.5, and BA.2.86/JN.1 with descendants KP.2, KP.3, and XFG. XBB.1.5 is a recombinant lineage.
(TIF)

S2 Fig Nanosota-9A binds the JN.1 spike ectodomain via a cross-interacting mechanism [33]. (A) Previously determined cryo-EM structure of the JN.1 spike ectodomain complexed with Nanosota-9A (PDB 9CO8). (B) Two Nanosota-9A molecules cross-interact with two JN.1 RBDs (one standing up and one lying down). Each RBD contains a core and a receptor-binding motif (RBM). This 2:2 binding mode creates a main interface between Nanosota-9A and the RBM of one RBD and a minor interface between Nanosota-9A and the core of the other RBD. Human antibodies cannot fit into the Nanosota-9A binding epitope on the lying-down RBD. (C) Neutralizing potency of Nanosota-9A-Fc (Fc-tagged Nanosota-9A) against different Omicron subvariants.
(TIF)

S3 Fig Characterization of two Nanosota-9B candidates. (A) Structure-guided evolution of Nanosota-9A yielded two candidates (Nanosota-9B-1 and Nanosota-9B-2), both of which bind the KP.3 spike ectodomain. They differ at four residues targeted for randomization. (B) Binding of each candidate to the KP.3 spike ectodomain was assessed by ELISA. Plates were coated with His-tagged KP.3 ectodomain, incubated with HA-tagged Nanosota-9B, and binding was detected with anti-HA antibodies. Nanosota-9B-1 showed stronger binding and was therefore selected as Nanosota-9B for further characterization.
(TIF)

S4 Fig Flowchart of cryo-EM image processing and 3D reconstruction for the KP.3 spike ectodomain/Nanosota-9B complex. Representative raw micrographs and 2D class averages are shown. 3D refinements using particles from high-quality 3D classes yielded a 3.06 Å map. Subsequent local refinement improved the density of the bound nanobody. The angular distribution plot, final maps, half-map FSC curves, and local-resolution estimates are enclosed in the dashed black boxes.
(TIF)

S5 Fig Superposition of Nanosota-9A and Nanosota-9B. Nanosota-9A (cyan; PDB ID 9CO9) and Nanosota-9B (yellow) are structurally highly similar, with differences confined to engineered residues. Substitutions at positions 101 and 102 induce small local conformational changes in the corresponding loop, whereas substitutions at positions 31 and 32 do not. Selected residues are shown as sticks.
(TIF)

S6 Fig Nanosota-9A/9B-Fc shows low neutralizing potency against the Omicron subvariant XFG. (A) Neutralization of Omicron pseudoviruses by Nanosota-9A/9B-Fc, performed as in Fig 3C. (B) Mapping of RBD residues mutated within or near the Nanosota-9 binding epitopes. (C) Sequence alignment of Nanosota-9-contacting RBD residues across Omicron subvariants. Residues in direct contact with Nanosota-9 are colored blue (conserved) or red (mutated). RBD residues that do not directly contact Nanosota-9 but are mutated from JN.1 to KP.2/KP.3/XFG are shown in bold black. Asterisks denote fully conserved positions, colons indicate strong conservation, and periods indicate weak conservation.
(TIF)

S1 Table Cryo-EM data collection, refinement, and validation statistics of the KP.3 spike ectodomain/Nanosota-9B complex. (PDF)

## References

[1] Cao Y, Wang J, Jian F, Xiao T, Song W, Yisimayi A, et al. Omicron escapes the majority of existing SARS-CoV-2 neutralizing antibodies. Nature. 2022;602(7898):657–63. doi: 10.1038/s41586-021-04385-3 35016194 PMC8866119

[2] Flemming A. Omicron, the great escape artist. Nat Rev Immunol. 2022;22(2):75. doi: 10.1038/s41577-022-00676-6 35017722 PMC8749340

[3] Planas D, Saunders N, Maes P, Guivel-Benhassine F, Planchais C, Buchrieser J, et al. Considerable escape of SARS-CoV-2 Omicron to antibody neutralization. Nature. 2022;602(7898):671–5. doi: 10.1038/s41586-021-04389-z 35016199

[4] Greaney AJ, Starr TN, Gilchuk P, Zost SJ, Binshtein E, Loes AN, et al. Complete Mapping of Mutations to the SARS-CoV-2 Spike Receptor-Binding Domain that Escape Antibody Recognition. Cell Host Microbe. 2021;29(1):44–57.e9. doi: 10.1016/j.chom.2020.11.007 33259788 PMC7676316

[5] Greaney AJ, Loes AN, Crawford KHD, Starr TN, Malone KD, Chu HY, et al. Comprehensive mapping of mutations in the SARS-CoV-2 receptor-binding domain that affect recognition by polyclonal human plasma antibodies. Cell Host Microbe. 2021;29(3):463–476.e6. doi: 10.1016/j.chom.2021.02.003 33592168 PMC7869748

[6] Ye G, Liu B, Li F. Cryo-EM structure of a SARS-CoV-2 omicron spike protein ectodomain. Nat Commun. 2022;13(1):1214. doi: 10.1038/s41467-022-28882-9 35241675 PMC8894419

[7] Ryman JT, Meibohm B. Pharmacokinetics of Monoclonal Antibodies. CPT Pharmacometrics Syst Pharmacol. 2017;6(9):576–88. doi: 10.1002/psp4.12224 28653357 PMC5613179

[8] Ovacik M, Lin K. Tutorial on Monoclonal Antibody Pharmacokinetics and Its Considerations in Early Development. Clin Transl Sci. 2018;11(6):540–52. doi: 10.1111/cts.12567 29877608 PMC6226118

[9] Könning D, Zielonka S, Grzeschik J, Empting M, Valldorf B, Krah S, et al. Camelid and shark single domain antibodies: structural features and therapeutic potential. Curr Opin Struct Biol. 2017;45:10–6. doi: 10.1016/j.sbi.2016.10.019 27865111

[10] De Meyer T, Muyldermans S, Depicker A. Nanobody-based products as research and diagnostic tools. Trends Biotechnol. 2014;32(5):263–70. doi: 10.1016/j.tibtech.2014.03.001 24698358

[11] Du L, Yang Y, Zhang X, Li F. Recent advances in nanotechnology-based COVID-19 vaccines and therapeutic antibodies. Nanoscale. 2022;14(4):1054–74. doi: 10.1039/d1nr03831a 35018939 PMC8863106

[12] Muyldermans S. Nanobodies: natural single-domain antibodies. Annu Rev Biochem. 2013;82:775–97. doi: 10.1146/annurev-biochem-063011-092449 23495938

[13] Steeland S, Vandenbroucke RE, Libert C. Nanobodies as therapeutics: big opportunities for small antibodies. Drug Discov Today. 2016;21(7):1076–113. doi: 10.1016/j.drudis.2016.04.003 27080147

[14] Romao E, Morales-Yanez F, Hu Y, Crauwels M, De Pauw P, Hassanzadeh GG, et al. Identification of Useful Nanobodies by Phage Display of Immune Single Domain Libraries Derived from Camelid Heavy Chain Antibodies. Curr Pharm Des. 2016;22(43):6500–18. doi: 10.2174/1381612822666160923114417 27669966

[15] Ye G, Gallant J, Zheng J, Massey C, Shi K, Tai W, et al. The development of Nanosota-1 as anti-SARS-CoV-2 nanobody drug candidates. Elife. 2021;10:e64815. doi: 10.7554/eLife.64815 34338634 PMC8354634

[16] Wu X, Cheng L, Fu M, Huang B, Zhu L, Xu S, et al. A potent bispecific nanobody protects hACE2 mice against SARS-CoV-2 infection via intranasal administration. Cell Rep. 2021;37(3):109869. doi: 10.1016/j.celrep.2021.109869 34644535 PMC8492916

[17] Nambulli S, Xiang Y, Tilston-Lunel NL, Rennick LJ, Sang Z, Klimstra WB, et al. Inhalable Nanobody (PiN-21) prevents and treats SARS-CoV-2 infections in Syrian hamsters at ultra-low doses. Sci Adv. 2021;7(22):eabh0319. doi: 10.1126/sciadv.abh0319 34039613 PMC8153718

[18] Schoof M, Faust B, Saunders RA, Sangwan S, Rezelj V, Hoppe N, et al. An ultrapotent synthetic nanobody neutralizes SARS-CoV-2 by stabilizing inactive Spike. Science. 2020;370(6523):1473–9. doi: 10.1126/science.abe3255 33154106 PMC7857409

[19] Ye G, Bu F, Pan R, Mendoza A, Yang G, Spiller B, et al. Structure-guided in vitro evolution of nanobodies targeting new viral variants. PLoS Pathog. 2024;20(9):e1012600. doi: 10.1371/journal.ppat.1012600 39325826 PMC11460708

[20] Li F. Structure, Function, and Evolution of Coronavirus Spike Proteins. Annual Review of Virology. 2016;3(1):237–61. doi: 10.1146/annurev-virology-110615-042301 27578435 PMC5457962

[21] Shang J, Wan Y, Luo C, Ye G, Geng Q, Auerbach A, et al. Cell entry mechanisms of SARS-CoV-2. Proc Natl Acad Sci U S A. 2020;117(21):11727–34. doi: 10.1073/pnas.2003138117 32376634 PMC7260975

[22] Geng Q, Shi K, Ye G, Zhang W, Aihara H, Li F. Structural Basis for Human Receptor Recognition by SARS-CoV-2 Omicron Variant BA.1. J Virol. 2022;96(8):e0024922. doi: 10.1128/jvi.00249-22 35343765 PMC9044962

[23] Bálint G, Vörös-Horváth B, Széchenyi A. Omicron: increased transmissibility and decreased pathogenicity. Signal Transduct Target Ther. 2022;7(1):151. doi: 10.1038/s41392-022-01009-8 35525870 PMC9077027

[24] Planas D, Staropoli I, Michel V, Lemoine F, Donati F, Prot M, et al. Distinct evolution of SARS-CoV-2 Omicron XBB and BA.2.86/JN.1 lineages combining increased fitness and antibody evasion. Nature Communications. 2024;15(1):2254. doi: 10.1038/s41467-024-46490-7 38480689 PMC10938001

[25] Lu Y, Ao D, He X, Wei X. The rising SARS-CoV-2 JN.1 variant: evolution, infectivity, immune escape, and response strategies. MedComm (2020). 2024;5(8):e675. doi: 10.1002/mco2.675 39081516 PMC11286544

[26] Planas D, Staropoli I, Planchais C, Yab E, Jeyarajah B, Rahou Y, et al. Escape of SARS-CoV-2 Variants KP.1.1, LB.1, and KP3.3 from Approved Monoclonal Antibodies. Pathog Immun. 2024;10(1):1–11. doi: 10.20411/pai.v10i1.752 39391808 PMC11464000

[27] Wang Q, Mellis IA, Ho J, Bowen A, Kowalski-Dobson T, Valdez R, et al. Recurrent SARS-CoV-2 spike mutations confer growth advantages to select JN.1 sublineages. Emerg Microbes Infect. 2024;13(1):2402880. doi: 10.1080/22221751.2024.2402880 39259045 PMC11407393

[28] Shang J, Ye G, Shi K, Wan Y, Luo C, Aihara H, et al. Structural basis of receptor recognition by SARS-CoV-2. Nature. 2020;581(7807):221–4. doi: 10.1038/s41586-020-2179-y 32225175 PMC7328981

[29] Wan Y, Shang J, Graham R, Baric RS, Li F. Receptor Recognition by the Novel Coronavirus from Wuhan: an Analysis Based on Decade-Long Structural Studies of SARS Coronavirus. J Virol. 2020;94(7):e00127–20. doi: 10.1128/JVI.00127-20 31996437 PMC7081895

[30] Zhang W, Shi K, Geng Q, Ye G, Aihara H, Li F. Structural basis for mouse receptor recognition by SARS-CoV-2 omicron variant. Proc Natl Acad Sci U S A. 2022;119(44):e2206509119. doi: 10.1073/pnas.2206509119 36256797 PMC9636943

[31] Zhang W, Shi K, Geng Q, Herbst M, Wang M, Huang L, et al. Structural evolution of SARS-CoV-2 omicron in human receptor recognition. J Virol. 2023;97(8):e0082223. doi: 10.1128/jvi.00822-23 37578233 PMC10506476

[32] Ye G, Bu F, Pan R, Mendoza A, Saxena D, Zheng J, et al. Dual-role epitope on SARS-CoV-2 spike enhances and neutralizes viral entry across different variants. PLoS Pathog. 2024;20(9):e1012493. doi: 10.1371/journal.ppat.1012493 39236072 PMC11407660

[33] Ye G, Bu F, Saxena D, Turner-Hubbard H, Herbst M, Spiller B, et al. Discovery of Nanosota-9 as anti-Omicron nanobody therapeutic candidate. PLoS Pathog. 2024;20(11):e1012726. doi: 10.1371/journal.ppat.1012726 39591462 PMC11630572

[34] Ye G, Pan R, Bu F, Zheng J, Mendoza A, Wen W. Discovery of Nanosota-2, -3, and -4 as super potent and broad-spectrum therapeutic nanobody candidates against COVID-19. J Virol. 2023;97(11):e0144823. doi: 10.1128/jvi.01448-23 37855638 PMC10688364

[35] Li F. Receptor recognition mechanisms of coronaviruses: a decade of structural studies. J Virol. 2015;89(4):1954–64. doi: 10.1128/JVI.02615-14 25428871 PMC4338876

[36] Vaskuri GJ, Ye G, Bu F, Yang D, Jonsson CB, Turner-Hubbard H, et al. Long-Circulating Nanobody Confers Durable Prophylaxis against Severe Acute Respiratory Syndrome Coronavirus 2 Omicron Infection. Adv Nanobiomed Res. 2025;5(8):2400214. doi: 10.1002/anbr.202400214 40851835 PMC12369981

[37] CDC. https://www.cdc.gov/covid/php/variants/variants-and-genomic-surveillance.html

[38] Piccoli L, Park Y-J, Tortorici MA, Czudnochowski N, Walls AC, Beltramello M, et al. Mapping Neutralizing and Immunodominant Sites on the SARS-CoV-2 Spike Receptor-Binding Domain by Structure-Guided High-Resolution Serology. Cell. 2020;183(4):1024–1042.e21. doi: 10.1016/j.cell.2020.09.037 32991844 PMC7494283

[39] Pinto D, Sauer MM, Czudnochowski N, Low JS, Tortorici MA, Housley MP, et al. Broad betacoronavirus neutralization by a stem helix-specific human antibody. Science. 2021;373(6559):1109–16. doi: 10.1126/science.abj3321 34344823 PMC9268357

[40] Barnes CO, Jette CA, Abernathy ME, Dam K-MA, Esswein SR, Gristick HB, et al. SARS-CoV-2 neutralizing antibody structures inform therapeutic strategies. Nature. 2020;588(7839):682–7. doi: 10.1038/s41586-020-2852-1 33045718 PMC8092461

[41] Guo L, Chen Z, Lin S, Yang F, Yang J, Wang L, et al. Structural basis and mode of action for two broadly neutralizing nanobodies targeting the highly conserved spike stem-helix of sarbecoviruses including SARS-CoV-2 and its variants. PLoS Pathog. 2025;21(4):e1013034. doi: 10.1371/journal.ppat.1013034 40215243 PMC12052392

[42] Atwell S, Ultsch M, De Vos AM, Wells JA. Structural plasticity in a remodeled protein-protein interface. Science. 1997;278(5340):1125–8. doi: 10.1126/science.278.5340.1125 9353194

[43] Matthews JM. Plasticity at Protein–Protein Interaction Interfaces. Encyclopedia of Biophysics. Springer Berlin Heidelberg. 2013:1886–8. 10.1007/978-3-642-16712-6_173

[44] Li F, Berardi M, Li W, Farzan M, Dormitzer PR, Harrison SC. Conformational states of the severe acute respiratory syndrome coronavirus spike protein ectodomain. J Virol. 2006;80(14):6794–800. doi: 10.1128/JVI.02744-05 16809285 PMC1489032

[45] Shan S, Luo S, Yang Z, Hong J, Su Y, Ding F, et al. Deep learning guided optimization of human antibody against SARS-CoV-2 variants with broad neutralization. Proc Natl Acad Sci U S A. 2022;119(11):e2122954119. doi: 10.1073/pnas.2122954119 35238654 PMC8931377

[46] Desautels TA, Arrildt KT, Zemla AT, Lau EY, Zhu F, Ricci D, et al. Computationally restoring the potency of a clinical antibody against Omicron. Nature. 2024;629(8013):878–85. doi: 10.1038/s41586-024-07385-1 38720086 PMC11111397

[47] Hsieh C-L, Goldsmith JA, Schaub JM, DiVenere AM, Kuo H-C, Javanmardi K, et al. Structure-based design of prefusion-stabilized SARS-CoV-2 spikes. Science. 2020;369(6510):1501–5. doi: 10.1126/science.abd0826 32703906 PMC7402631

[48] Geng Q, Wan Y, Hsueh F-C, Shang J, Ye G, Bu F, et al. Lys417 acts as a molecular switch that regulates the conformation of SARS-CoV-2 spike protein. Elife. 2023;12:e74060. doi: 10.7554/eLife.74060 37991488 PMC10695562

[49] Ridgway JB, Presta LG, Carter P. “Knobs-into-holes” engineering of antibody CH3 domains for heavy chain heterodimerization. Protein Eng. 1996;9(7):617–21. doi: 10.1093/protein/9.7.617 8844834

[50] Ye G, Bu F, Turner-Hubbard H, Herbst M, Du L, Yang G, et al. Structures of Marburgvirus glycoprotein and its complex with NPC1 receptor. Nature. 2026;653(8114):621–6. doi: 10.1038/s41586-026-10240-0 41813895 PMC13171430

[51] Zhang W, Shi K, Hsueh F-C, Mendoza A, Ye G, Huang L, et al. Structural basis for mouse receptor recognition by bat SARS2-like coronaviruses. Proc Natl Acad Sci U S A. 2024;121(32):e2322600121. doi: 10.1073/pnas.2322600121 39083418 PMC11317568

[52] Amanat F, White KM, Miorin L, Strohmeier S, McMahon M, Meade P, et al. An In Vitro Microneutralization Assay for SARS-CoV-2 Serology and Drug Screening. Curr Protoc Microbiol. 2020;58(1):e108. doi: 10.1002/cpmc.108 32585083 PMC7361222

[53] Punjani A, Rubinstein JL, Fleet DJ, Brubaker MA. cryoSPARC: algorithms for rapid unsupervised cryo-EM structure determination. Nat Methods. 2017;14(3):290–6. doi: 10.1038/nmeth.4169 28165473

[54] Rubinstein JL, Brubaker MA. Alignment of cryo-EM movies of individual particles by optimization of image translations. J Struct Biol. 2015;192(2):188–95. doi: 10.1016/j.jsb.2015.08.007 26296328

[55] Rohou A, Grigorieff N. CTFFIND4: Fast and accurate defocus estimation from electron micrographs. J Struct Biol. 2015;192(2):216–21. doi: 10.1016/j.jsb.2015.08.008 26278980 PMC6760662

[56] Emsley P, Cowtan K. Coot: model-building tools for molecular graphics. Acta Crystallogr D Biol Crystallogr. 2004;60(Pt 12 Pt 1):2126–32. doi: 10.1107/S0907444904019158 15572765

[57] Adams PD, Afonine PV, Bunkóczi G, Chen VB, Davis IW, Echols N, et al. PHENIX: a comprehensive Python-based system for macromolecular structure solution. Acta Crystallogr D Biol Crystallogr. 2010;66(Pt 2):213–21. doi: 10.1107/S0907444909052925 20124702 PMC2815670

