# Supplementary figures and images for: Update and reuse: Structure-guided nanobody evolution against SARS-CoV-2 escape
Source: PLoS Pathog. 2026 May 18;22(5):e1014223. doi: 10.1371/journal.ppat.1014223 (PMC13193604; doi:10.1371/journal.ppat.1014223)

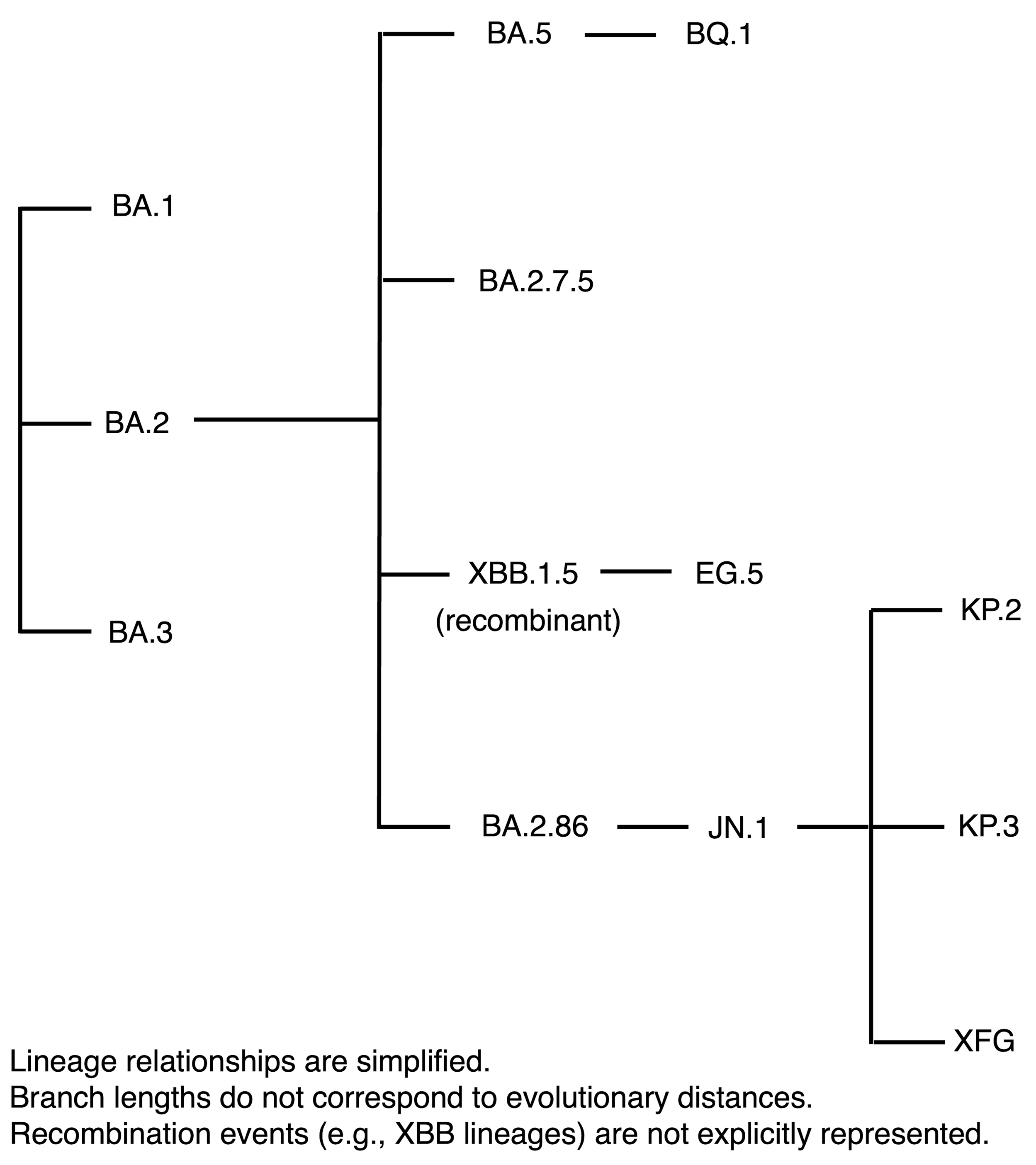

Supplement: S1 Fig — This diagram is not intended as a phylogenetic tree. Shown are BA.1, BA.2, BA.3, BA.5/BQ.1, BA.2.7.5, XBB.1.5/EG.5, and BA.2.86/JN.1 with descendants KP.2, KP.3, and XFG. XBB.1.5 is a recombinant lineage. (TIF) [file ppat.1014223.s001.tif]

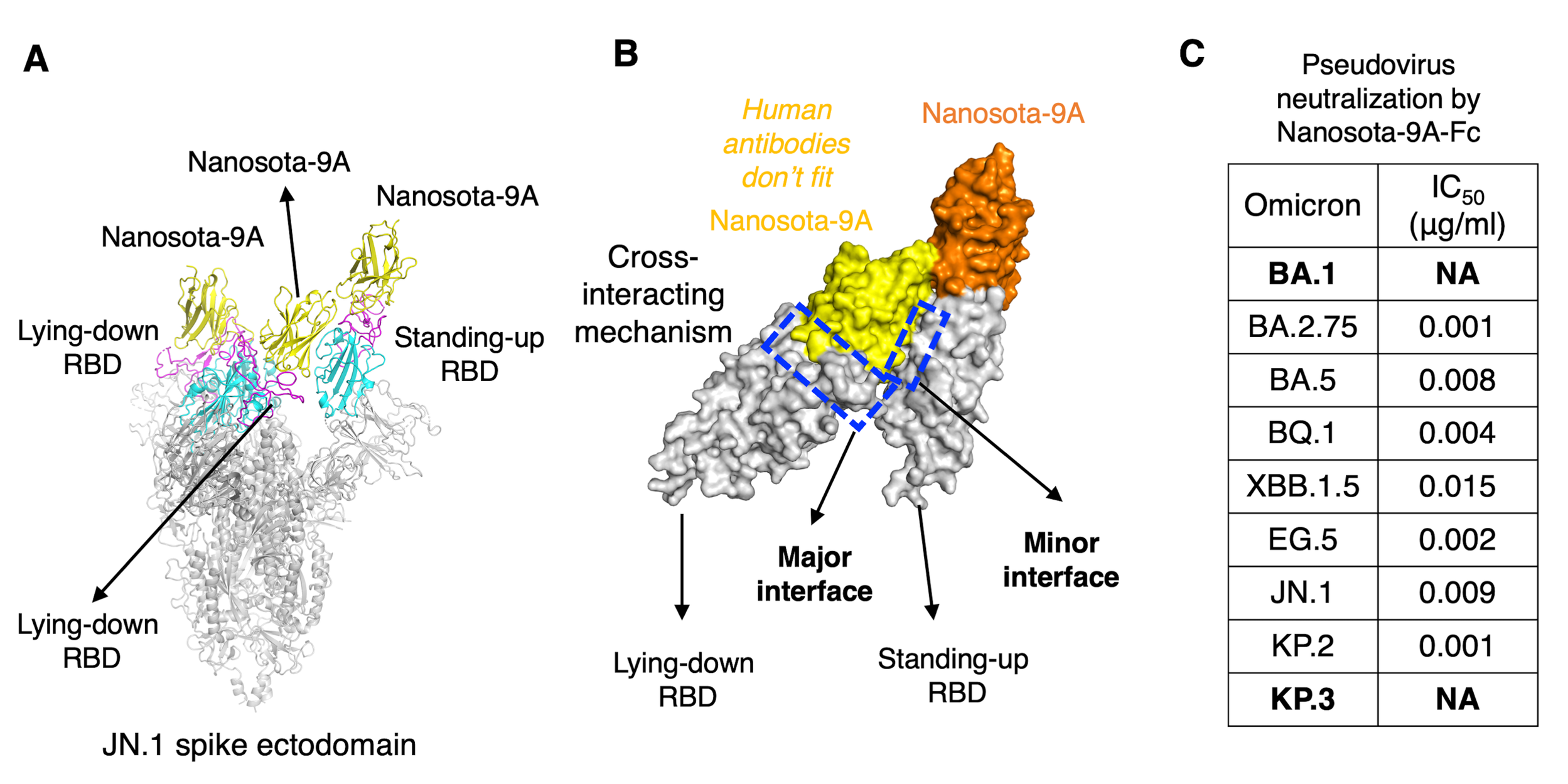

Supplement: S2 Fig — (A) Previously determined cryo-EM structure of the JN.1 spike ectodomain complexed with Nanosota-9A (PDB 9CO8). (B) Two Nanosota-9A molecules cross-interact with two JN.1 RBDs (one standing up and one lying down). Each RBD contains a core and a receptor-binding motif (RBM). This 2:2 binding mode creates a main interface between Nanosota-9A and the RBM of one RBD and a minor interface between Nanosota-9A and the core of the other RBD. Human antibodies cannot fit into the Nanosota-9A binding epitope on the lying-down RBD. (C) Neutralizing potency of Nanosota-9A-Fc (Fc-tagged Nanosota-9A) against different Omicron subvariants. (TIF) [file ppat.1014223.s002.tif]

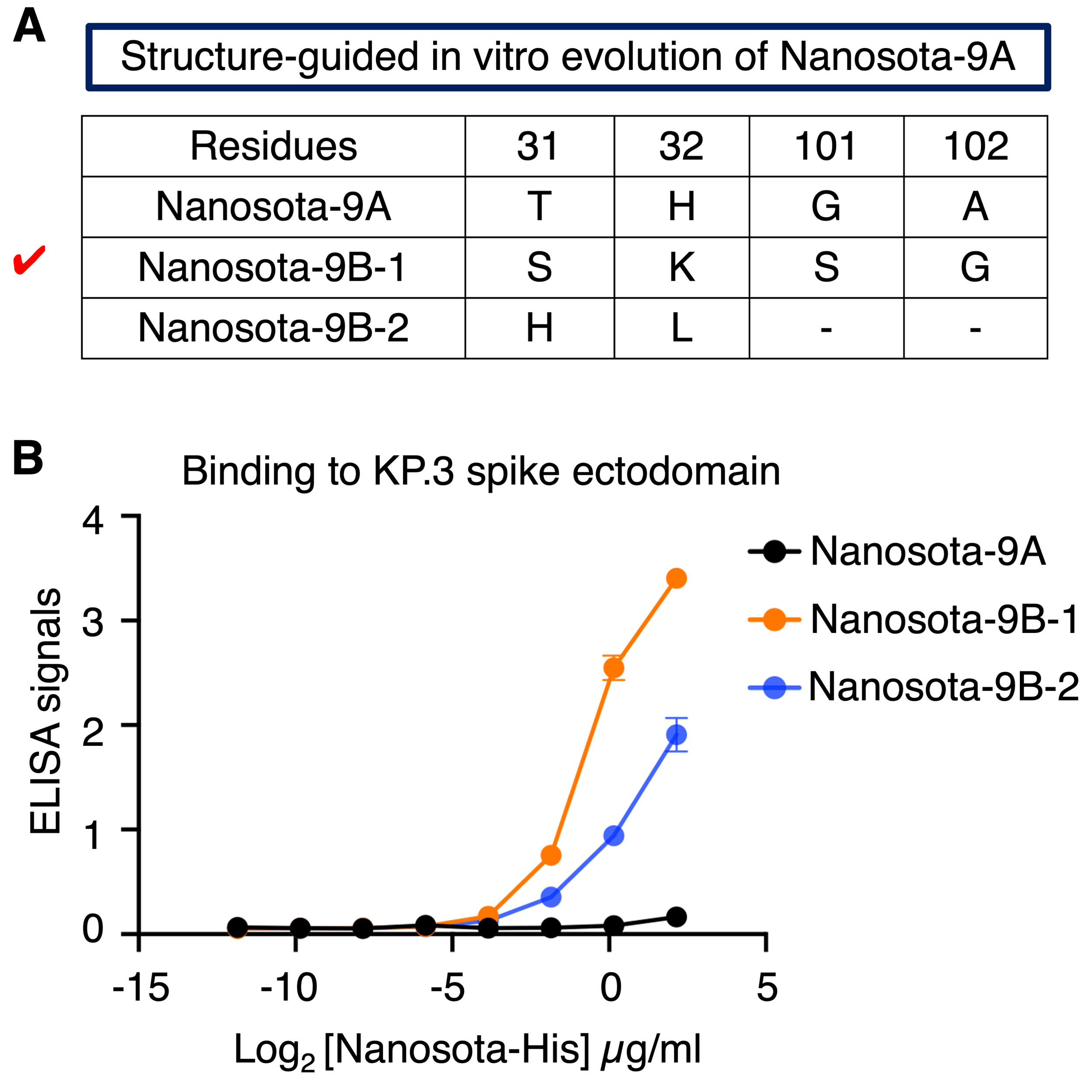

Supplement: S3 Fig — (A) Structure-guided evolution of Nanosota-9A yielded two candidates (Nanosota-9B-1 and Nanosota-9B-2), both of which bind the KP.3 spike ectodomain. They differ at four residues targeted for randomization. (B) Binding of each candidate to the KP.3 spike ectodomain was assessed by ELISA. Plates were coated with His-tagged KP.3 ectodomain, incubated with HA-tagged Nanosota-9B, and binding was detected with anti-HA antibodies. Nanosota-9B-1 showed stronger binding and was therefore selected as Nanosota-9B for further characterization. (TIF) [file ppat.1014223.s003.tif]

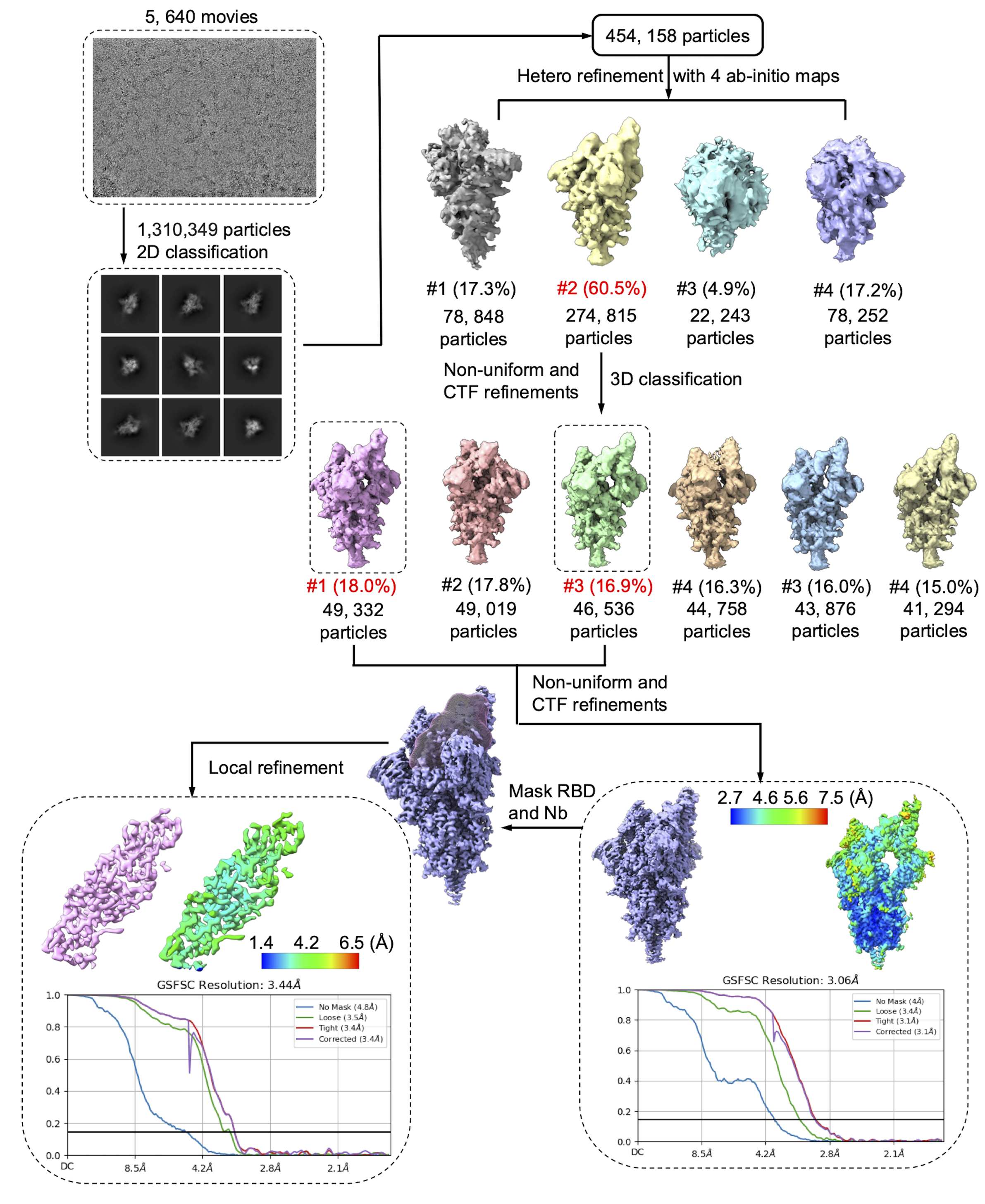

Supplement: S4 Fig — Representative raw micrographs and 2D class averages are shown. 3D refinements using particles from high-quality 3D classes yielded a 3.06 Å map. Subsequent local refinement improved the density of the bound nanobody. The angular distribution plot, final maps, half-map FSC curves, and local-resolution estimates are enclosed in the dashed black boxes. (TIF) [file ppat.1014223.s004.tif]

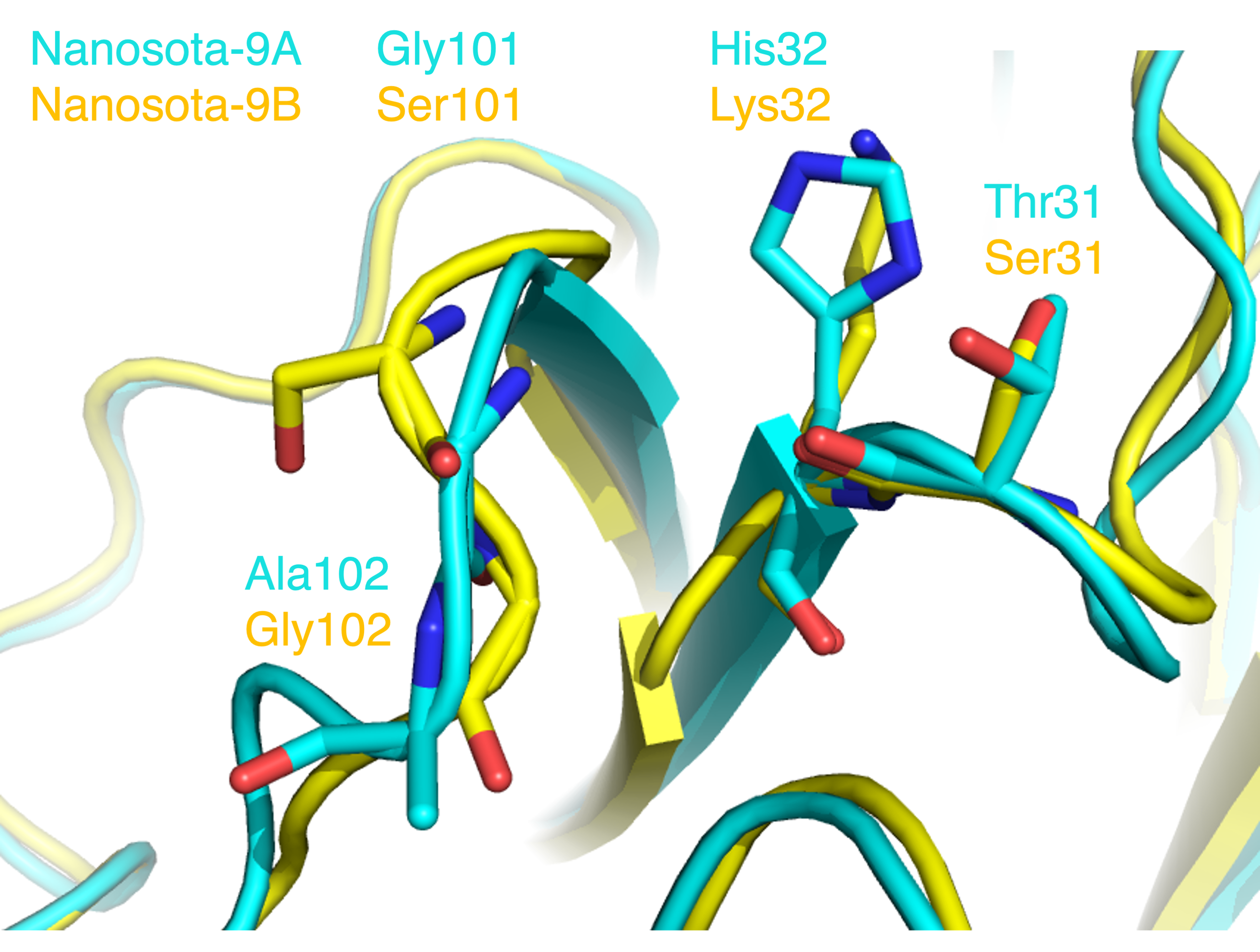

Supplement: S5 Fig — Nanosota-9A (cyan; PDB ID 9CO9) and Nanosota-9B (yellow) are structurally highly similar, with differences confined to engineered residues. Substitutions at positions 101 and 102 induce small local conformational changes in the corresponding loop, whereas substitutions at positions 31 and 32 do not. Selected residues are shown as sticks. (TIF) [file ppat.1014223.s005.tif]

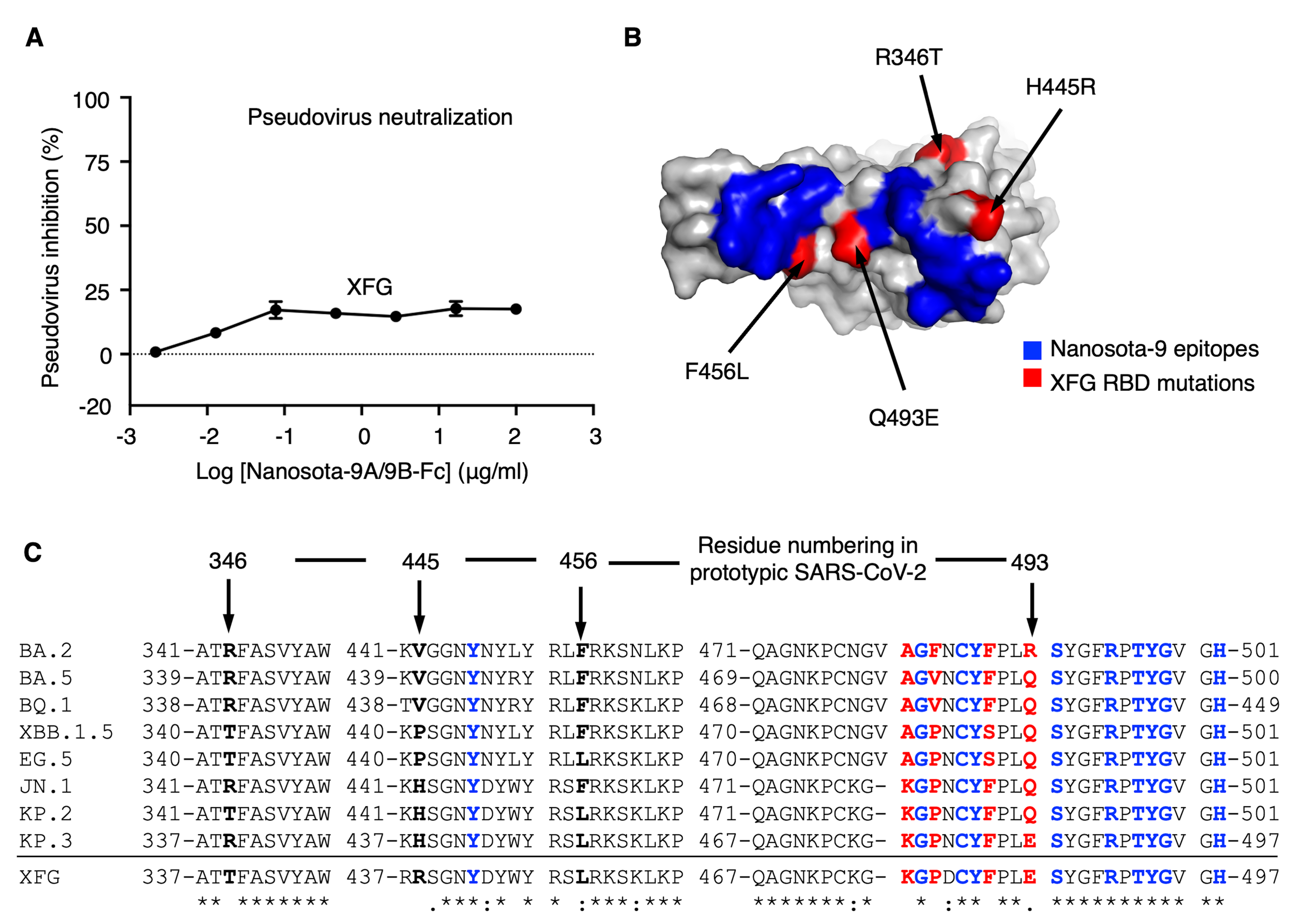

Supplement: S6 Fig — (A) Neutralization of Omicron pseudoviruses by Nanosota-9A/9B-Fc, performed as in Fig 3C. (B) Mapping of RBD residues mutated within or near the Nanosota-9 binding epitopes. (C) Sequence alignment of Nanosota-9-contacting RBD residues across Omicron subvariants. Residues in direct contact with Nanosota-9 are colored blue (conserved) or red (mutated). RBD residues that do not directly contact Nanosota-9 but are mutated from JN.1 to KP.2/KP.3/XFG are shown in bold black. Asterisks denote fully conserved positions, colons indicate strong conservation, and periods indicate weak conservation. (TIF) [file ppat.1014223.s006.tif]
