# Supplementary material for: Update and reuse: Structure-guided nanobody evolution against SARS-CoV-2 escape
Source: PLoS Pathog. 2026 May 18;22(5):e1014223. doi: 10.1371/journal.ppat.1014223 (PMC13193604; doi:10.1371/journal.ppat.1014223)
Supplement: S1 Table — (PDF) [file ppat.1014223.s007.pdf]

Table S1: Cryo-EM data collection, refinement and validation statistics.

	KP.3 spike ectodomain/ Nanosota-9B complex	KP.3 spike ectodomain/ Nanosota-9B complex after local refinement
**Data collection and processing**		
Magnification	130,000	130,000
Voltage (kV)	300	300
Electron exposure (e–/Å^2)	51.2	50
Defocus range (μm)	-0.75 ~ -2.5	-0.75 ~ -2.5
Pixel size (Å)	0.664	0.664
Symmetry imposed	C1	C1
Initial particle images (no.)	1,310,349	1,310,349
Final particle images (no.)	95,868	95,868
Map resolution (Å)	3.06	3.44
FSC threshold	0.143	0.143
Map resolution range (Å)	1.4-6.5	2.5-7.8
**Refinement**		
Initial model used (PDB code)	9CO8	9CO8
Model resolution (Å)	3.3	3.7
FSC threshold	0.5	0.5
Model resolution range (Å)	3.0-57.1	1.8-22.3
Map sharpening *B* factor (Å^2)	69.3	97.5
Model composition		
Non-hydrogen atoms	28753	3098
Protein residues	3633	395
Ligands	26	0
B factors (Å^2)		
Protein	144.34	96.36
Nucleotide		
Ligand	145.41	
R.m.s. deviations		
Bond lengths (Å)	0.005	0.005
Bond angles (°)	0.912	0.926
Validation		
MolProbity score	1.56	1.42
Clashscore	3.55	2.31
Poor rotamers (%)	0.03	0.00
Ramachandran plot		
Favored (%)	93.77	93.86
Allowed (%)	5.99	6.14
Disallowed (%)	0.25	0.00
